# Preliminary study on fabrication, characterization and synergistic anti-lung cancer effects of self-assembled micelles of covalently conjugated celastrol–polyethylene glycol–ginsenoside Rh2

**DOI:** 10.1080/10717544.2017.1326540

**Published:** 2017-05-22

**Authors:** Peng Li, XiaoYue Zhou, Ding Qu, Mengfei Guo, Chenyi Fan, Tong Zhou, Yang Ling

**Affiliations:** 1 Department of Oncology, Changzhou Cancer Hospital of Soochow University, Changzhou, P.R. China,; 2 Affiliated Hospital of Integrated Traditional Chinese and Western Medicine, Nanjing University of Chinese Medicine, Nanjing, P.R. China,; 3 Jiangsu Province Academy of Traditional Chinese Medicine, Nanjing, P.R. China, and; 4 Clinical Oncology Laboratory, Changzhou Cancer Hospital of Soochow University, Changzhou, P.R. China

**Keywords:** Celastrol, ginsenoside Rh2, micelle, anti-lung cancer, drug release

## Abstract

The aim of this study was to develop an amphipathic polyethylene glycol (PEG) derivative that was bi-terminally modified with celastrol and ginsenoside Rh2 (Celastrol-PEG-G Rh2). Such derivative was capable of forming novel, celastrol-loaded polymeric micelles (CG-M) for endo/lysosomal delivery and thereby synergistic treatment of lung cancer. Celastrol-PEG-G Rh2 with a yield of 55.6% was first synthesized and characterized. Its critical micellar concentration was 1 × 10^−5 ^M, determined by pyrene entrapment method. CG-M had a small particle size of 121.53 ± 2.35 nm, a narrow polydispersity index of 0.214 ± 0.001 and a moderately negative zeta potential of –23.14 ± 3.15 mV. Celastrol and G Rh2 were rapidly released from CG-M under acidic and enzymatic conditions, but slowly released in normal physiological environments. In cellular studies, the internalization of celastrol and G Rh2 by human non-small cell lung cancer (A549) cells treated with CG-M was 5.8-fold and 1.8-fold higher than that of non-micelle control. Combinational therapy of celastrol and G Rh2 using CG-M exhibited synergistic anticancer activities in cell apoptosis and proliferation assays via rapid drug release within endo/lysosomes. Most importantly, the celastrol in CG-M exhibited a long elimination half-life of 445.3 ± 43.5 min and an improved area under the curve of 645060.8 ± 63640.7 ng/mL/h, that were 1.03-fold and 2.44-fold greater than those of non-micelle control, respectively. These findings suggest that CG-M is a promising vector for precisely releasing anticancer drugs within the tumor cells, and thereby exerts an improved synergistic anti-lung cancer effect.

## Introduction

A polymeric micelle is a core-shell structure formed spontaneously from amphiphilic polymers in aqueous phase via hydrophobic forces (Kwon & Okano, 1996). The hydrophilic segments of the polymers form the shell, whereas the lipophilic segments form the hydrophobic drug reservoir (Felber et al., 2012). Similar to other drug delivery systems (such as liposomes, nanoparticles and microemulsions), polymeric micelles have become one of the most widely used drug delivery systems in the field of nano-sized drug delivery (Wong et al., 2010). In general, polymeric micelles exhibit the following characteristics and advantages: (1) The hydrophobic interactions between the hydrophobic segments and poorly soluble drugs impart a relatively high drug-loading capacity (DLC) (van Nostrum, 2004); (2) Polymeric micelles have a lower critical micelle concentration (CMC) and higher thermodynamic and kinetic stability than small-molecule micelles do, suggesting that their structures remain intact in blood circulation with enhanced permeability and retention (EPR) effects (Lu et al., 2013); (3) They have improved functional flexibility, where active tumor-targeting capacity can be enhanced by introducing tumor-specific ligands or chemical modifications can render them responsive to pH, contact surface, redox potential, or heat (Gong et al., 2013); and (4) Polymeric micelles are relatively nontoxic, non-immunogenic polymers that do not readily accumulate in the body and are easily metabolized (Desale et al., 2013).

Celastrol is a triterpenoid compound extracted from the root of *Tripterygium wilfordii* that exhibits significant antiproliferative effects via various mechanisms on non-small cell lung carcinoma (NSCLC) cells (such as A549) (Aqil et al., 2016; Niemelä et al., 2015; Kang et al., 2013), breast cancer cells (such as MCF-7 and MDA-MB-231) (Kim et al., 2013; Mi et al., 2014) and liver cancer cells (such as HepG2) (Han et al., 2014). Thus, celastrol is a promising antineoplastic candidate from natural sources. However, there is still no commercially available celastrol formulation for clinical applications so far, mainly due to the following two reasons: (1) It has a low bioavailability due to its poor water solubility; and (2) It can cause severe hepatotoxicity, nephrotoxicity and immunotoxicity due to its nonspecific distribution *in vivo*. Hence, the establishment of a hydrophilic celastrol delivery system with higher efficacy and reduced toxicity has become an important issue that needs to be addressed urgently. The encapsulation technique, using an amphiphilic polymeric material, could prevent the aforementioned drawbacks. For example, the antitumor drug paclitaxel encapsulated in polymers, such as polyethylene glycol (PEG)-poly(aspartic acid) and PEG-poly(D,L-lactide), has been marketed (Kim et al., 2001; Negishi et al., 2006; Matsumura, 2008), and a doxorubicin-loaded, Pluronic L61/F127-mixed micelle preparation (SP1049C) has entered the clinical trial stage (Danson et al., 2004; Valle et al., 2011). These formulations exhibit a significantly reduced systemic toxicity with significantly improved bioavailability, stability and targeted distribution.

Many Chinese clinical doctors prescribe traditional Chinese medicines (e.g., ginseng, *Ganoderma lucidum* and *Cordyceps sinensis*) to act as synergistic agents with chemotherapeutics, such as doxorubicin, cis-platinum and paclitaxel (Xu et al., 2016). For example, ginsenoside Rh2 (G Rh2) is a compound derived from the traditional Chinese medicine, ginseng, that exhibits a synergistic antitumor effect by regulating the immune function of cancer patients. It is a widely recognized active ingredient, which is capable of alleviating the side effects of chemotherapeutics (Li et al., 2011; An et al., 2013). Considering this, the combination treatment with two or more synergistic drugs is exhibiting increasing potential in the field of antitumor therapy.

Although polymeric micelles encapsulating two drugs simultaneously to achieve a synergistic antitumor effect have been reported, there are at least three issues that need to be addressed: (1) Stably encapsulating two drugs with different physicochemical properties into one micelle system, (2) Releasing the drugs with the desired pharmacokinetics and (3) Releasing the active ingredients specifically at the target site. The synergistic effect will be greatly reduced if co-delivery cannot be achieved. In addition to encapsulating antitumor components via hydrophobic interactions, covalent conjugation between insoluble drugs and hydrophilic macromolecules to form new amphiphilic polymers that self-assemble in aqueous phase to form micellar drug delivery systems is also a promising drug delivery approach (Lu et al., 2014).

Herein, we report the development of a polymeric material with a three-section structure, “hydrophobic-hydrophilic-hydrophobic”, through the covalent conjugation of celastrol and G Rh2 onto both ends of PEG segments via ester linkages. Celastrol was encapsulated in the hydrophobic core of the micelles using the principle of “similarity and intermiscibility.” We expect that this drug delivery system will persist over a long time in the blood circulation and will be internalized into endosomes after accumulating at the tumor site via the EPR effect. This drug delivery system is expected to release a large amount of celastrol and G Rh2, thus enabling synergistic antitumor effects. In this study, we mainly verify the rationality and efficacy of this drug delivery system with respect to its structural characteristics, *in vitro* anti-lung tumor effects and *in vivo* pharmacokinetics.

## Methods

### Materials

Celastrol (purity >99.9%) was purchased from Sinopharma group Co., Ltd. (Shanghai, China). G Rh2 (purity >98%) was purchased from Nangjing zelang biotech Co., Ltd. (Jiangsu, China). Bis(4-nitrophenylcarbonate) polyethylene glycol (5 kDa, pNP-PEG-pNP) was purchased from Xiamen SINOPEG BioTech Co. (Xiamen, China). Pyrene was purchased from Invitrogen Co. (Carlsbad, CA). Coumarin 6 was provided by Sigma Co. (UK). 3-(4,5-Dimethylthiazol-2-yl)-2,5-diphenyltetrazolium bromide (MTT) was obtained from Aladdin Chemical Co. (Shanghai, China). RPMI-1640 medium, penicillin–streptomycin solution and fetal bovine serum (FBS) were provided by Thermo Fisher Scientific Inc. (Beijing, China). Water in this study was prepared using Elix®5 Milli Q-water purification system (Millipore, MA). All other chemicals and reagents were analytical grade.

### pNP-PEG-celastrol synthesis

PEGylated celastrol was synthesized referring to previously reported methods for chemical modification onto both ends of the PEG segment (Torchilin et al., 2001a,b). Firstly, 2.04 g dehydrated pNP-PEG-pNP was dissolved in 20 mL of toluene and refluxed for 30 min. The solvent was evaporated under reduced pressure to obtain 1.92 g of dried white powder. After that, 45.2 mg of celastrol (equivalent to 0.1 mmol) was dissolved in 5 mL of anhydrous chloroform and 2% (wt%) anhydrous triethylamine was slowly added dropwise with magnetic stirring at room temperature. After 20 min, 532.6 mg of pNP-PEG-pNP (equivalent to 0.1 mmol) was added, and the mixture was slowly heated to reflux for 24 h. Last, the crude product was precipitated using cold ether. After removing the solvent under vacuum, 459.7 mg of white solid (pNP-PEG-celastrol) was obtained with 81.6% yield. Additionally, Celastrol-PEG-G Rh2 with different molar ratio of celastrol and G Rh2 was synthesized through the above-mentioned method, but adjustment for the feeding ratio of reactants.

### G-Rh2-PEG-celastrol synthesis

A total of 31.5 mg of G Rh2 (equivalent to 0.05 mmol) was dissolved in 30 mL of anhydrous chloroform. Two equivalents of sodium carbonate were added, followed by stirring at room temperature for 15 min. After heating the mixture at 60 °C for 20 min, the anhydrous chloroform containing 281.7 mg of pNP–PEG–celastrol (equivalent to 0.05 mmol) was added dropwise to refluxed for 24 h (Torchilin et al., 2001a,b). Afterward, the mixture was concentrated under vacuum, followed by precipitation using a mixed solution of ether and ethanol (8:1, v/v). A total of 208.0 mg of white solid powder was obtained with a yield of 68.1%. The synthetic route of G-Rh2-PEG-celastrol was list in Figure S1.

### Characterization of the chemical structure of G-Rh2-PEG-celastrol

Ten milligrams of G-Rh2-PEG-celastrol was dissolved in 0.6 mL of deuterated chloroform for the detection of chemical shifts using proton nuclear magnetic resonance (^1^H NMR) spectroscopy (400 M, Bruker AVANCE III HD, Karlsruhe, Germany). Twenty milligrams of G-Rh2–PEG–celastrol was mixed and compressed into a pellet with KBr, followed by detecting specific functional groups using Fourier transform infrared spectroscopy (FT-IR, Nicolet IS-10, ThermoFisher, MA).

### *CMC of celastrol-PEG-G Rh2* (Qu et al., 2013)

One-hundred microliters of 3 × 10^−5 ^M pyrene in acetone was added to a clean injection vial to evaporate the acetone, followed by the addition of 5 mL of different concentrations (1 × 10^−5^–1 mg/mL) of Celastrol-PEG-G Rh2 aqueous solution. The final concentration of pyrene was 6 × 10^−7 ^M, which was lower than its solubility limit in water. The mixture was sonicated at room temperature for 30 min, incubated at 65 °C in a water bath for 3 h, cooled to room temperature overnight, and then filtered. The excitation spectrum of sample solution was measured at an excitation wavelength range of 300–360 nm and an emission wavelength (λ_em_) of 390 nm with a slit width of 3 nm. The excitation spectrum of pyrene was red-shifted with increased concentration of the analyte, and the excitation wavelength of each concentration was recorded. The ratio of the fluorescence intensity corresponding to the excitation wavelength before and after the red shift was plotted against the concentration of the sample. When the ratio changed significantly, the concentration was taken as the CMC.

### Preparation and characterization of micelles

#### Preparation

Two milligrams of celastrol and 10.0 mg of Celastrol-PEG-G Rh2 were dissolved in 200 μL of anhydrous ethanol and slowly added dropwise into 5.0 mL of deionized water with magnetic stirring until the solution appeared transparent and opalescence. The solution was then loaded into a dialysis bag with a molecular-weight cutoff (MWCO) of 3 kDa and dialyzed in flowing water for 8 h to obtain the Celastrol-PEG-G Rh2 micelles (CG-M). A total of 50 μL of 500 μM coumarin 6 (C6) was used in place of 2.0 mg of celastrol to obtain the C6 fluorescent-labeled micelles (C6/CG-M) as described above. A blank CG-M (CG-M_blank_) was also prepared as described above, without the addition of celastrol.

#### Characterization

The freshly prepared CG-M was diluted in methanol and quantified by high-performance liquid chromatography (HPLC) to calculate the encapsulation efficiency (EE) of celastrol using the following formula: EE (%) = sample concentration × dilution factor × volume/feeding amount × 100. The particle size, polydispersity index (PDI) and zeta potential were measured using dynamic light scattering (DLS). The CG-M was loaded onto a copper mesh and stained with 1% phosphomolybdic acid, followed by infrared drying, prior to the observation of particle morphology under a transmission electron microscope (TEM, Tecnai 12, FEI, OR).

### Assessment of CG-M stability and drug release

One-hundred microliters of freshly prepared CG-M was diluted 10-fold with phosphate-buffered (PB) solutions of different pH values (4.5, 5.5, 6.5 and 7.4) and incubated for 30 min, prior to the measurement of particle sizes and zeta potentials of micelles. CG-M was diluted 4-fold, 9-fold, 99-fold and 999-fold with deionized water to measure the particle size and zeta potential. Likewise, CG-M solution was suspended in an equivalent volume of fetal bovine serum (FBS) to measure the micellar size and zeta potential after 1–12 h of incubation.

Two milliliters of freshly prepared CG-M were loaded into a dialysis tubing with MWCO of 3 kDa, which was then immersed in 200 mL of various PB solutions with 50% (v%) rat serum and/or different pH values (5.0, 6.5 and 7.4). When the *in vitro* dissolution apparatus was set to an agitation rate of 60 r^.^min^−1^ and a temperature of 37 °C, the amount of celastrol and G-Rh2 in the medium at determined time intervals was measured using HPLC (Agilent1200, Agilent, CA) to calculate the corresponding hydrolytic and release rates. The chromatographic conditions of celastrol were as follows (Su et al., 2014a): the mobile phase was methanol/2% acetic acid (90/10, v/v), with a detection wavelength at 426 nm and the detection conditions for G Rh2 were according to a previous publication (Gu et al., 2006), the mobile phase was methanol/acetonitrile/water (8/8/1, v/v) and the wavelength was 203 nm. Both drugs were passed through a C18 column (4.6 mm × 150 mm, 5-μm particles, Agilent) with a flow rate of 1.0 mL/min.

### Study of CG-M at the cellular level

#### Culture of A549 cells

The human NSCLC cell line, A549, was cultured in RPMI 1640 medium containing 10% FBS at 37 °C and 5% CO_2_ in an incubator with saturated humidity. The medium was replaced every 2 days and the cells were passaged at the split ratio of 1:8 every 5–7 days. Cells grown to the logarithmic growth phase were harvested for further experiments.

#### Antiproliferative effect by MTT assay

The A549 cells were seeded in a 96-well plate at 5 × 10^3^ cells/well and incubated for 24 h. The medium was then discarded and each well received 100 μL of different concentrations of CG-M, CG-M_blank_, celastrol + G Rh2, celastrol and G Rh2. After 48 h of incubation, 100 μL of 0.5 mg/mL MTT in phosphate-buffered saline (PBS) was added to each well, followed by 4 h of incubation in the dark. The medium was then discarded and 100 μL of dimethyl sulfoxide was added to each well. The plate was shaken at low speed for 3 min, and the absorbance at 570 nm was measured using a microplate reader to calculate cell viability. Cell viability (%) = Absorbance of the drug treatment group (A_drug_)/Absorbance of the control group (A_control_) × 100. The IC_50_ was calculated by GraphPad prism 6.0 software (GraphPad Software Inc., San Diego, CA). The combined index (CI) was calculated with the following formula: CI = IC_50_ of celastrol in combination group/IC_50_ of celastrol + IC_50_ of G Rh2 in combination group/IC_50_ of G Rh2 (Qu et al., 2017; Wu et al., 2017), which represents a synergistic antitumor effect if the value is less than 1.

#### Cellular uptake

The freshly prepared CG-M, CG-M_blank_ and celastrol + G Rh2 solutions were adjusted to 100 μM, and stored until needed. The A549 cells were cultured as adherent cells in a 24-well plate until reaching 70% confluence. The medium was then discarded, and 400 μL of the above solutions was added individually to each well, followed by 2 h of incubation at 37 °C. The drug solutions were then discarded and each well was rinsed three times with 500 μL of PBS per rinse to remove the residual drug. Next, sodium dodecyl sulfate (SDS) cell lysis buffer (160 μL per well) was added, and the plate was incubated at 37 °C for 2 min. The resulting lysate was centrifuged at 8000 g for 5 min. Twenty microliters of the supernatant was taken to measure protein content using a BCA protein assay kit (ThermoFisher, Beijing, China), and 100 μL of the supernatant was deproteinized by adding 100 μL of methanol, vortexing for 5 min and clearing by centrifugation at 8000 *g* for 5 min. The amount of drug in the supernatant was then measured by HPLC. The cellular uptake was calculated using the following formula. Cellular uptake (μg/mg) = Q_drug in cells_/Q_cells protein_, where Q_drug in cells_ and Q_cells protein_ represent the intracellular drug content and protein content, respectively.

#### Intracellular transport mechanism

A total of 1 × 10^5^ A549 cells was seeded in a confocal microscopy dish (NEST Biotechnology, Beijing, China) and grown until 60% confluence, followed by 2 h and 4 h of incubation with C6/CG-M. The medium was then removed and the cells were rinsed 5 times with 0.5 mL ice-cold PBS each to remove the residual C6. The cells were then stained with 0.5 mL of 100 nM LysoTracker Red (KeyGen BioTECH, Jiangsu, China) at 37 °C in the dark for 30 min and then rinsed thoroughly with ice-cold PBS. The adherent cells were fixed with 10% paraformaldehyde for 30 min, rinsed with PBS and the intracellular fluorescent distribution was observed by laser confocal microscopy.

#### Induction of cell apoptosis

A total of 1 × 10^5^ A549 cells was seeded in a 24-well plate and grown until 80% confluence. Four hundred microliters of CG-M, CG-Mblank, celastrol + G Rh2, celastrol (20 μM), or G Rh2 (8 μM) solutions were added to each well. After 8 h of incubation, the cells were rinsed thoroughly with ice-cold PBS and digested with 200 μL of trypsin to harvest the cell suspension, which was then transferred (50 μL) to a 96-well plate and incubated with an equal volume of Annexin V-PE Apoptosis detection kit (Guava, Merck-Millipore, MA) in the dark for 15 min. Cell apoptosis was measured by flow cytometry (Guava 6HT).

### Pharmacokinetic studies

Twelve male Sprague Dawley rats weighing 200 ± 20 g were randomly divided into 3 treatment groups as follows: (1) C + G; (2) CG-M and (3) CG-M_blank_. After tail vein injection of the micelle formulation at a celastrol dose of 3 mg/kg, approximately 500 μL of blood was collected from the plexus venous in the eye at specific time intervals, and the plasma was collected after centrifugation (3000 rpm for 10 min). The critical pharmacokinetic parameters were determined by Kinetica 4.4 software (Thermo, MA), including area under the plasma concentration − time curve (AUC_0-∞_), half-life (*t*_1/2_), the maximal drug concentration (C_max_), the time of the C_max_ observed (*T*_max_) and mean residence time (MRT). In this experiment, all operations for the animals were carried out in accordance with the protocol approved by the animal ethics committee at our institution.

## Results and discussion

### Physiochemical characterization of celastrol–PEG–G Rh2

#### ^1^H NMR confirmation

**Figure 1.**
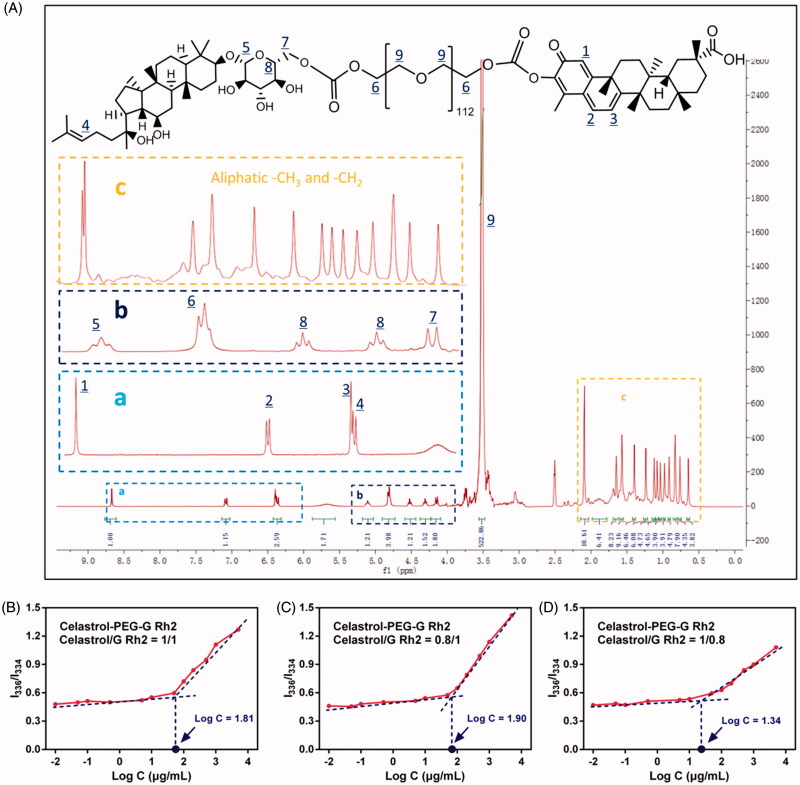
(A) The ^1^H NMR spectrum of Celastrol-PEG-G Rh2. The labeled peaks represent the corresponding signal of hydrogen in the chemical structure. The determination of CMC for Celastrol-PEG-G Rh2 with different molar ratios, (B) 1:1, (C) 0.8:1 and (D)1:0.8, between celastrol and G Rh2.

Generally, the H^+^ of the phenolic hydroxyl group in the structure of celastrol is more susceptible to ionization and more acidic than the conventional phenolic hydroxyl group due to the surrounding conjugated system, thus, it is favorable to esterification. Likewise, the 6-hydroxyl (6-OH) group on the G Rh2 glycosyl fragment also exhibits a better chemical modifiability and can be acylated to form esters. Based on these chemical properties, Celastrol–PEG–G Rh2 is capable of being synthesized in an anhydrous, basic organic phase via covalent conjugation between the nitrophenoxy groups of pNP–PEG–pNP, phenolic hydroxyl group of celastrol and the hydroxyl groups on the G Rh2 glycosylated fragment. The total yield of the product was 55.6%. Structural identification using ^1^H NMR (see Figure 1A, frame a) showed signals between 8.6 and 6.4 ppm that were characteristic of the aromatic fragment of celastrol, and the integral ratio of the other H was consistent with the theoretical value, but signal for the hydrogen of the phenolic hydroxyl group had disappeared, consistent with celastrol conjugation to the PEG fragment. The signals at 6.3 ppm (see Figure 1A, frame a) and 5.1-4.1 ppm (see Figure 1A, frame b) were assigned to the alkene double bond and glycosyl fragment of G Rh2, respectively. The signals between 0.8 and 2.1 ppm (see frame c) were assigned to the large numbers of hydrogens in the steroidal rings, but the 6-OH signal had disappeared. In addition, there was a typical signal of PEG between 3.2 and 3.5 ppm, indicating that the two drugs had been conjugated to both ends of the PEG segments. More importantly, the characteristic hydrogens on celastrol and G Rh2 had an integral ratio of about 1:1, consistent with celastrol, PEG and G Rh2 having the same molar ratio in the structure of the product. ^1^H NMR (d_6_-DMSO) δ 8.67 (s, 1H), 7.07 (d, 1H), 6.40 (d, 1H), 6.36 (t, 1H), 5.13 (t, 1H), 4.83 (t, 4H), 4.54–4.33 (t, 2H), 4.16 (d, 2H), 3.52–3.20 (s, 522H), 2.15–0.84 (s, 78H).

#### Ftir

In order to confirm that G Rh2 and celastrol had been covalently conjugated to both ends of the PEG segments, we also performed FTIR analysis to identify the target compound. It can be seen from Figure S2 that the characteristic peak of the benzene ring at both sides of pNP-PEG-pNP (about 1600 cm^−1^) disappeared, while there is a distinctive peak at 1738.3 cm^−1^ that is assigned to the characteristic ester carbonyl group at the linkage site of Celastrol–PEG–G Rh2. The characteristic peak of C = C vibration in the G Rh2 fragment is also observed at 1636.3 cm^−1^. The obvious peak at 1149.6 cm^−1^ can be assigned to the C-O bond in the PEG fragment. In addition, the glycosyl hydroxyl peak in the structure can be observed in the > 2880 cm^−1^ region. Therefore, we further confirm from this spectrum that the compound is the target compound designed in this study.

#### Determination of the CMC of celastrol-PEG-G Rh2

The CMC of Celastrol-PEG-G Rh2 was determined using the encapsulated hydrophobic fluorescent probe, pyrene. The excitation spectrum of pyrene was located at 334 nm when the concentration of Celastrol-PEG-G Rh2 was lower than its CMC. The fluorescence intensity and excitation wavelength of pyrene were essentially unchanged with a gradual increase in Celastrol–PEG–G Rh2 concentration. However, the fluorescence intensity of pyrene increased drastically with a shift of excitation wavelength to 336 nm when the concentration of Celastrol–PEG–G Rh2 was higher than its CMC. The concentration logarithm (Log(C)) was plotted against the ratio of fluorescence intensity at 336 nm to that at 334 nm (I_336_/I_334_), and the concentration corresponding to the intersection point of the two lines was taken as the CMC of Celastrol-PEG-G Rh2 (Qu et al., 2013). As shown in Figure 1(B), this concentration was approximately 64.6 μg/mL (1 × 10^−5 ^M). This indicated that the self-assembled Celastrol–PEG–G Rh2 micelles exhibit a greater anti-dilution capability in water after the conjugation of hydrophobic structural fragments to both ends of the PEG, which is similar to the conclusion drawn by Wang et al. (Wang et al., 2014), who constructed an octavinyl silsesquioxane–PEG–PLA amphiphilic block copolymer with a CMC value of 1.5 × 10^−5 ^M. In addition, we found that the CMC value can be affected by the mol ratio of celastrol to G Rh2 in the Celastrol-PEG-G Rh2 structure, where the CMC value decreased with increasing proportion of celastrol (see Figure 1C and 1D). Generally, a reduction of CMC leads to an increase in micellar particle size (Lin et al., 2003), which is not favorable for realizing the EPR effect. Therefore, we used Celastrol-PEG-G Rh2 (celastrol/G Rh2, 1/1, mol/mol) as the carrier material in further studies.

### Preparation of CG-M

**Figure 2.**
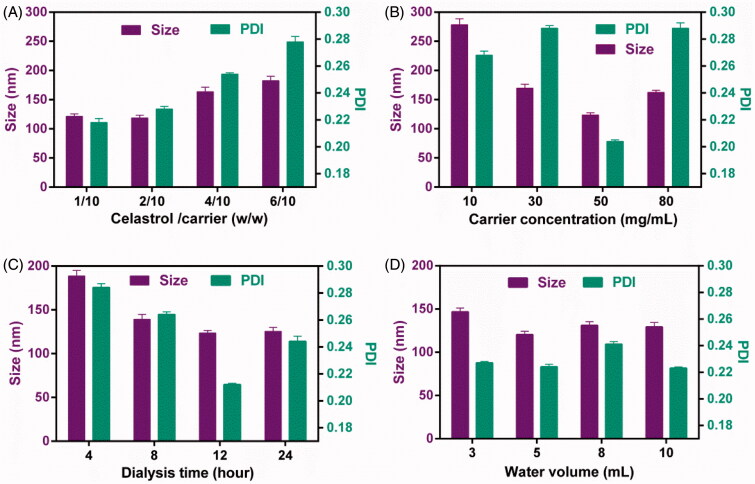
The influence of (A) the weight ratio of celastrol to carrier, (B) the carrier concentration, (C) the dialysis time and (D) the water volume on the particle size and PDI of CG-M. All data are presented as mean ± SD (*n* = 4).

In this study, CG-M was prepared by the ethanol injection-dialysis method, and the important factors affecting the physiochemical properties of CG-M include the drug-carrier ratio, carrier concentration, volume of deionized water and duration of dialysis (Qu et al., 2009). The effect of the above-mentioned factors on the preparation of CG-M was evaluated using a single factor test method. As shown in Figure 2(A), the micellar size was only 125 nm and the PDI was approximately 0.22 when the drug-carrier ratio was (1 ∼ 2)/10. As the value was increased to 6/10, the micellar size increased dramatically to 175 nm, with a significant increase in PDI, suggesting that drug overload increases the micellar size and PDI (Kataoka et al., 2001). The concentration of the carrier in ethanol also represents the amount of ethanol loaded into the same carrier, which may significantly affect its capability to form micelles. Figure 2(B) shows that the micellar size was approximately 270 nm when the concentration of the carrier was 10 mg/mL, but as the concentration increased to 50 mg/mL, the micellar size decreased significantly to approximately 110 nm with a significant decrease in the PDI. However, the micellar size decreased slightly as the concentration increased to 80 mg/mL. These results indicate that 50 mg/mL of carrier is more favorable for preparing CG-M with a minimum particle size and PDI. The duration of dialysis also influences the mean particle size and PDI, because of the amount ethanol residue in the micelles. As shown in Figure 2(C), 4 and 12 h of dialysis resulted in particle sizes of 185 and 120 nm, respectively, but the latter particles had a lower PDI, suggesting that the ethanol can be completely removed after 12 h of dialysis. Figure 2(D) demonstrates the effect of dispersion medium volume on the micellar size, where the micellar size and PDI did not change significantly when the volume was greater than 3 mL. In order to prepare CG-M with the smallest size and PDI, we have optimized the preparation process to use a drug-carrier ratio of 2/10, carrier concentration of 50 mg/mL, duration of dialysis of 12 h and dispersion medium volume of 5 mL.

### Characterization of CG-M

**Figure 3.**
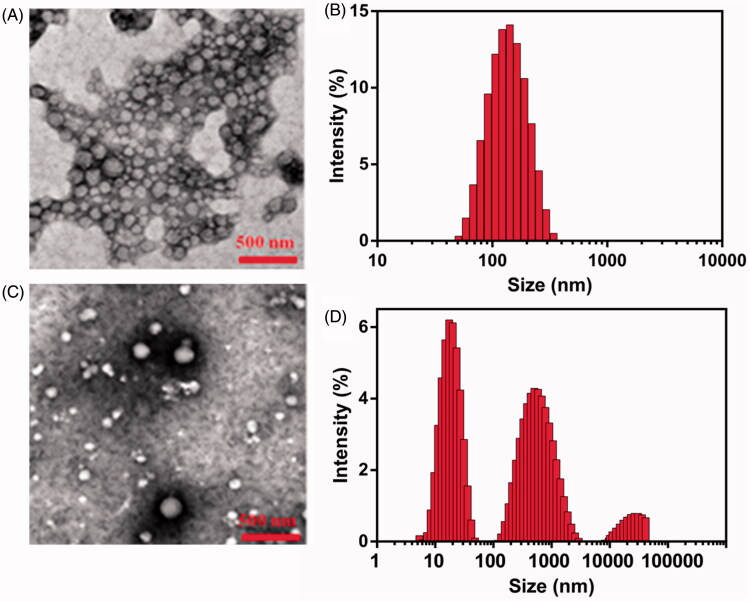
Morphology studies. (A) The morphology of CG-M in the FBS-free PBS studied by TEM, (B) the hydrodynamic size of CG-M in the FBS-free PBS measured by DLS, (C) the morphology of CG-M studied by TEM after incubation with the 50% (v%) FBS for 12 h and (D) the hydrodynamic size of CG-M measured by DLS after 12 h incubation with the 50% (v%) FBS.

The important indicators of CG-M, such as size, PDI, zeta potential and EE, are listed in Table 1. The size and PDI of CG-M_blank_ were 106.41 ± 2.96 nm and 0.244 ± 0.003, respectively. The surface of CG-M has a medium negative charge, which might be associated with the carboxyl group of the celastrol exposed on the outside of CG-M. The particle size of CG-M increased slightly to 121.53 ± 2.35 nm after being loaded with a small amount of celastrol, but the PDI and zeta potential did not change significantly, suggesting that only the particle size of micelles would change after being loaded with a certain mass of hydrophobic drugs. Because of celastrol as the hydrophobic core, the EE of celastrol of CG-M reached 85.23 ± 4.38%. As shown in Figure 3(A), the morphology of CG-M with a Celastrol–PEG–G Rh2 concentration of 0.1 mM was uniform, spherical and evenly dispersed, without obvious precipitation of water-insoluble drugs. The size of most particles dispersed into PB (pH 7.4) ranged between 90 and 120 nm, which was consistent with the particle size distribution (PSD) of DLS (Figure 3B). On the contrary, there were large amounts of precipitated crystals with significantly dispersed PSD after the CG-M was incubated with FBS-containing PB for 12 h (Figure 3C and D), which could be related to the precipitation of hydrophobic drugs released from disassembled CG-M because of the degradation by serum esterase.

**Table 1.** Characterization of CG-M loaded with different amounts of celastrol (*n* = 3).

Formulation	Size (nm)	PDI	Zeta (mV)	EE (%)
CG-M_blank_	106.41 ± 2.96	0.244 ± 0.003	−21.45 ± 2.94	NA
CG-M	121.53 ± 2.35	0.214 ± 0.001	−23.14 ± 3.15	85.23 ± 4.38

### Stability evaluation

**Figure 4.**
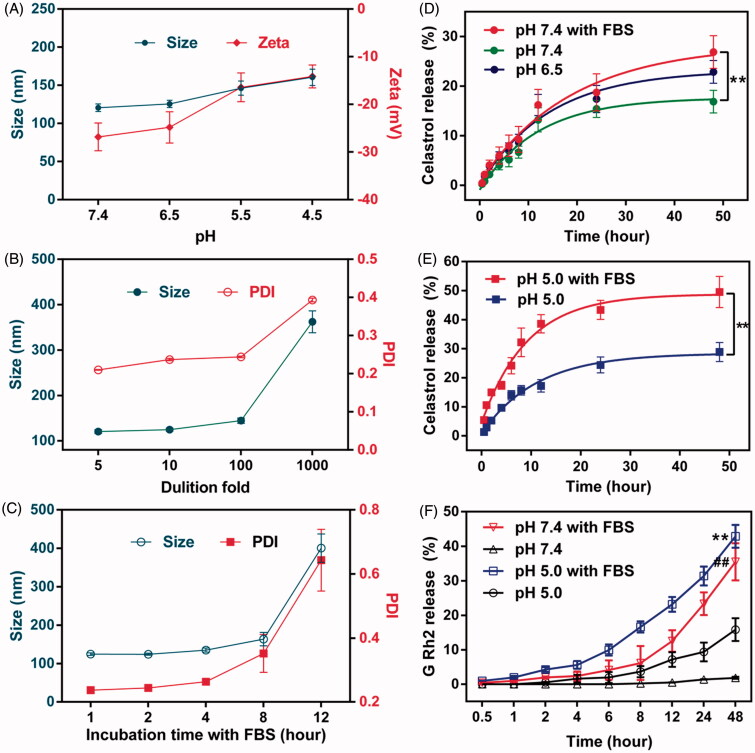
Evaluation on stability and drug release. (A) The changes in the particle size and zeta potential of CG-M under different pH environments, (B) the changes in the particle size and PDI of CG-M under various dilutions, (C) the changes in the particle size and PDI of CG-M after incubation with the 50% (v%) FBS for 12 h, (D) accumulative release rate of celastrol from CG-M at pH 7.4, pH 7.4 + 50% FBS and pH 6.5 within 48 h, (E) accumulative release rate of celastrol from CG-M at simulated endosomal environment within 48 h and (F) accumulative release rate of G Rh2 from CG-M_blank_ at various pH and simulated physiologic environments within 48 h. **p < 0.01 versus pH 5.0; ##*p* < 0.01 versus pH 7.4. All the data are presented as mean ± SD (*n* = 3).

The stability of CG-M under the influence of various factors needs to be evaluated as it may encounter various pH environments, dilution by large volumes of body fluid and hydrolysis by various types of esterase *in vivo*. As shown in Figure 4(A), the particle size and zeta potential of CG-M at pH 7.4 were approximately 120 nm and –26 mV, respectively. However, the micellar size increased gradually to 160 nm and the zeta potential increased to –14 mV as the pH decreased. We inferred that this was associated with the release of celastrol and G Rh2 from Celastrol–PEG–G Rh2 due to acid-catalyzed hydrolysis of ester linkages. The accelerated release of drug in acidic tumor microenvironments is, therefore, crucial for the antitumor effect of CG-M. Additionally, CG-M may disassemble when diluted in a large volume of bodily fluid. Thus, we also examined its anti-dilution capability. Figure 4(B) shows that CG-M has a great anti-dilution capability, as its micellar size and PDI did not change significantly after dilution by 100-fold. Furthermore, nonspecific binding with serum proteins may also negatively affect the delivery of CG-M to the tumor (Peng & Mu, 2016). In this study, CG-M was incubated with FBS for 1∼12 h to observe changes of its size and zeta potential. As shown in Figure 4(C), the micellar size and PDI did not change significantly after being incubated with FBS for 8 h, but elevated drastically after 12 h. This trend was consistent with the results of TEM, further suggesting that the CG-M may have been partially disassembled with leakage of the hydrophobic drugs.

### Evaluation of drug release behavior

The key to the antitumor effect of CG-M *in vivo* is good stability in blood circulation, rapid disassembly within the tumor environment and a sufficient release of drugs in the cell cytoplasm (Biswas et al., 2017). In this experiment, we investigated the drug release or hydrolytic behavior of CG-M in an acidic tumor microenvironment (pH 6.5), normal physiological condition (pH 7.4), endo/lysosomes (pH 5.0), simulated blood (pH 7.4 + FBS) and simulated endo/lysosomes (pH 5.0 + FBS) (Gao et al., 2017; Chen et al., 2017). The results in Figures 4(D) and 4(E) show that the 48-h cumulative release rate of celastrol from CG-M was ∼20% under various pH conditions in the absence of FBS, suggesting that the micellar structure is relatively stable in the absence of esterase activity. In contrast, FBS significantly improved the drug release rate of CG-M at pH 7.4 and 5.0, where the latter resulted in a 48 h-cumulative release rate of nearly 50%. We also investigated the drug release curve of G Rh2 from CG-M_blank_ under different conditions to further confirm the involvement of FBS in the hydrolysis of ester linkages in the polymeric material. As shown in Figure 4(F), FBS significantly enhanced the hydrolytic release of G Rh2 under both pH conditions, but there were differences between the two conditions. Approximately 6.1% of G Rh2 was released from the CG-M_blank_ incubated in PBS medium at pH 7.4 for 8 h, indicating that the blank micelles can remain relatively stable after 8 h in blood circulation. Compared with that of FBS-free medium, the release rate of G Rh2 in FBS medium of pH 7.4 was significantly accelerated, with a 48 h-cumulative release rate of nearly 35%. In addition, the hydrolytic release rate of G Rh2 from CG-M was higher in FBS solution at pH 5.0 than that of the (pH 5.0 + FBS) group, with 24 h and 48 h-cumulative release rates of 31.4 and 42.8%, respectively. The results indicate that micelles can release a large amount of celastrol and G Rh2 for a synergistic antitumor effect after being internalized into the endo/lysosomes of tumor cells. The aforementioned results validated that the CG-M we designed is capable of remaining stable in the blood circulation and then rapidly releasing the bioactive ingredients within acidic subcellular organelles for a synergistic antitumor effect.

### Cellular studies of CG-M

**Figure 5.**
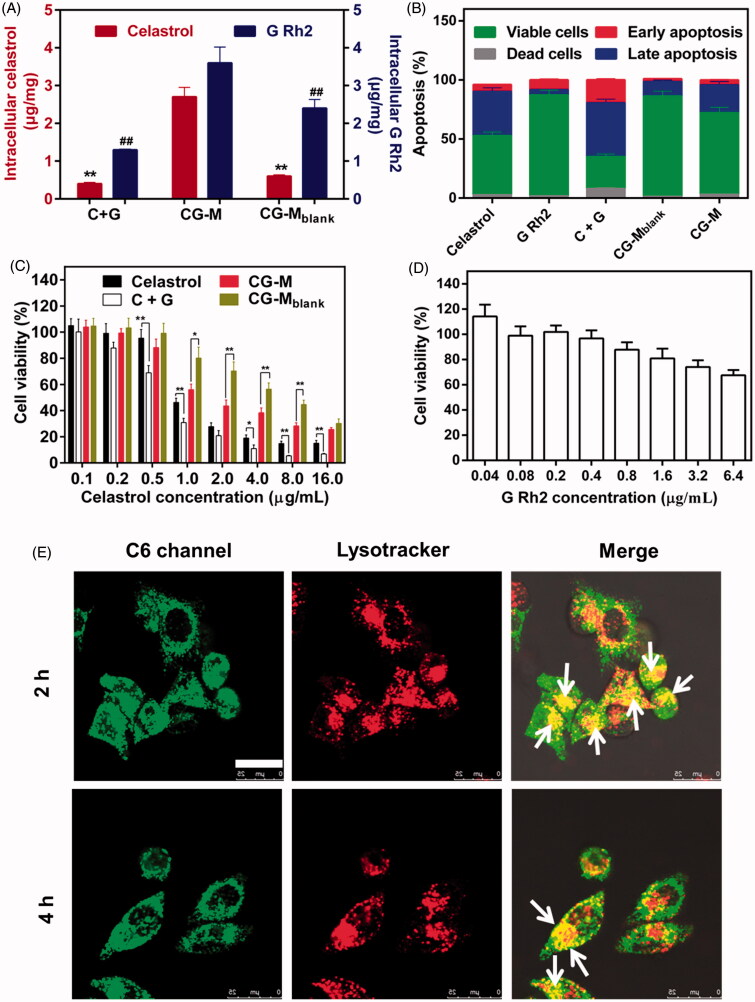
Cellular studies. (A) Cellular uptake of celastrol and G Rh2 by A549 cells, data are presented as mean ± SD (*n* = 4). **p < 0.01 versus celastrol of CG-M; #*P* < 0.01 versus G Rh2 of CG-M. (B) Apoptosis induction of A549 cells after treatment with different formulations, data are presented as mean ± SD (*n* = 4). The antiproliferative activity of (C) different celastrol formulations and (D) G Rh2 solution against A549 cells for 48 h, data are presented as mean ± SD (*n* = 6). **P < 0.01, *P < 0.05. (E) Intracellular delivery of C6/CG-M within A549 cells observed using CLSM. The arrows represent the micelles entrapped in the endo/lysosomes. Scale bar: 25 μm. The endo/lysosomes were stained by Lysotracker Red.

The antitumor effects of nano-fabrications are mainly determined by several factors, such as cellular uptake, induction of apoptosis and drug release (Zhao et al., 2016). In general, nanoscale drug delivery systems enter the cells via clathrin-mediated or caveolae-mediated endocytosis (Su et al., 2014b). Thus, they are expected to exhibit marked tumor-killing and apoptosis-inducing effects due to their greater uptake in tumor cells compared with that of free water-insoluble drugs. The antitumor effect of CG-M at the cellular level was evaluated using cellular uptake, induction of apoptosis and cytotoxicity as indicators. Figure 5(A) shows that CG-M released 2.7 μg/mg of celastrol and 3.6 μg/mg of G Rh2 into A549 cells after 2 h of incubation, with a significantly higher cellular uptake than that for C + G, which demonstrates the advantages of CG-M as a nanoscale drug delivery system with regard to tumor cell uptake. At the same time, the celastrol cellular uptake of CG-M was about 4.5-fold that of CG-M_blank_, indicating that the active ingredients in the polymeric material were not completely hydrolyzed, but the difference in cellular uptake was significantly reduced over time (data not shown). Figure 5(B) demonstrates the induction of apoptosis after the tumor cells were incubated with each group for 8 h. Among the groups, C + G could induce apoptosis in 48.7% of the cells, which was higher than the sum of apoptosis induced by celastrol and G Rh2 alone. However, the CG-M and CG-M_blank_ groups induced apoptosis in only 26.4 and 13.6% of the cells, respectively, which were significantly lower than that of the C + G group. A possible reason for these differences was that the induction of apoptosis depends on the amount of free drugs that enter the cells. Although CG-M has a greater ability to enter the cells than C + G does, it needs time to release a sufficient amount of drugs to induce apoptosis. Importantly, celastrol, C + G (5/2, w/w, corresponding to the weight ratio of celastrol to G Rh2 in CG-M) and CG-M_blank_ were taken as control groups to examine the antiproliferative effect of CG-M_blank_ on tumor cells after 48 h of incubation. C + G was found to exhibit a significantly greater antiproliferative effect than celastrol alone when the concentration of celastrol was higher than 0.5 μg/mL (see Figure 5C), and the calculated CI value of C + G was 0.91, indicating that the combination of two drugs provides a synergistic antiproliferative effect against A549 cells *in vitro*. The antitumor activity of both CG-M_blank_ and CG-M showed significant changes when the concentration of celastrol was higher than 1 μg/mL, suggesting that the antitumor effect of CG-M was mainly derived from the release of its encapsulated drugs at this concentration. However, there was no statistically significant difference in the antiproliferative effect between CG-M_blank_ and CG-M when the concentration was 16 μg/mL, indicating that the blank micelles can be catalyzed by intracellular hydrolases to release a sufficient amount of celastrol and G Rh2 for a synergistic effect at this concentration. Additionally, the IC_50_ of celastrol in C + G and CG-M groups were 0.62 ± 0.04 and 1.85 ± 0.14 μg/mL, respectively. The C + G group showed a greater tumor cell-killing effect than the CG-M group did, as the drug delivery system needed a longer hydrolytic time to release sufficient amounts of antitumor active ingredients. Figure 5(D) shows that the survival rate of cells was 67.5% when the concentration of G Rh2 was 6.4 μg/mL (equivalent to 16 μg/mL of celastrol in C + G), which further confirmed the synergistic antitumor effect of G Rh2 and celastrol.

The CG-M particles exhibit the greatest ability to release drugs under the conditions of pH 5.0 + FBS, indicating that this type of micelle has better drug efficacy if it can enter the endosomes or lysosomes. Hence, our study employed a fluorescence-staining co-localization technique to identify the endocytosed micelles as green and the intracellular lysosomes as red. The overlap of green and red fluorescence, observed as yellow fluorescence, represents C6/CG-M that was incorporated into the lysosomes after entering the cells. In contrast, the cells will be separately stained green and red if there was no lysosomal uptake (Tan et al., 2014). As shown in Figure 5(E), the cells were stained yellow over a wide area after being incubated with the micelles for 2 and 4 h, suggesting that the micelles were incorporated into the lysosomes, thereby facilitating the hydrolysis of polymeric material to release a large amount of drugs within A549 cells for a synergistic antitumor effect.

### Evaluation of pharmacokinetics

**Figure 6.**
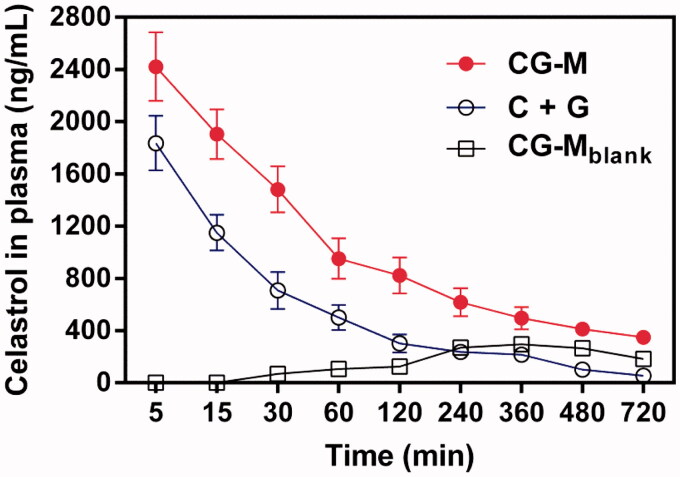
The concentration of celastrol in the plasma after intravenous injection of various celastrol formulations at dose of 3 mg/kg within 720 min, data are presented as mean ± SD (*n* = 4).

To validate whether CG-M was capable of circulating in the blood steadily for a long time due to the PEG segments, we compared the pharmacokinetic characteristics of various celastrol formulations. As shown in Figure 6, the peak plasma concentrations of celastrol in C + G and CG-M treatment groups were 1836.3 ± 209.2 and 2421.6 ± 285.2 ng/mL at the first time point, respectively. Over time, the plasma celastrol level of rats treated with the non-micelle (physically mixed) group (i.e., C + G) dropped rapidly until 120 min post injection. By comparison, the elimination rate of plasma celastrol with CG-M was significantly slower than that of the C + G group, suggesting an obvious advantage of PEG introduction. As we expected, CG-M_blank_ showed a modest but sustained release profile after 240 min post-injection, indicating that the polymeric carrier had a high stability in the physiological environment. As presented in Table 2, the AUC_0-∞_ of CG-M was 645060.8 ± 63640.7 ng/mL/h, which was 2.44-fold higher than that of the C + G group. Notably, the t_1/2_ and the MRT of CG-M were both exceptional among all the treatments. Interestingly, we found that the AUC_0-∞_ of CG-M_blank_ was higher than the physically mixed group, further validating the significance of celastrol-loaded polymeric micelles with respect to pharmacokinetics. All results provide concrete evidence that CG-M could greatly improve the bioavailability of free celastrol, as well as prolong blood circulation, to afford a more effective EPR effect and more potent synergistic antitumor efficacy.

**Table 2.** Pharmacokinetic parameters of celastrol formulations after i.v. administration at dose of 3 mg/kg (data are presented as mean ± SD, *n* = 4).

Parameter	CG-M	CG-M_blank_	C + G
Half-time (min)	445.3 ± 43.5^**^	251.1 ± 13.5	219.1 ± 13.5
MRT ^a^_0-∞_ (min)	606.2 ± 73.1^**^	410.2 ± 13.5	284.8 ± 19.3
AUC ^b^_0-∞_ (ng/mL/h)	645060.8 ± 63640.7^**^	292218 ± 2290.75	187501.6 ± 17924.7
C_max_ (ng/mL)	2421.6 ± 285.2^**^	294.3 ± 35.2	1836.3 ± 209.2
T_max_ (min)	5	360	5

^a^MRT _0-∞_, mean residence time; ^b^AUC _0-∞_, area under the plasma concentration–time curve.

** *p* < 0.01 versus C + G.

## Conclusion

In this study, we synthesized a novel drug vector, celastrol and G Rh2-conjugated PEG derivative, which was capable of loading celastrol to assemble an endo/lysosomal delivery micelle system. CG-M was spherical, homogeneous in size distribution and stable in physiologic environments, but readily disassembled and released drug under low pH and in the presence of serum hydrolases. In cellular studies, CG-M exhibited highly improved cellular uptake compared to that of control groups, strong induction of cell apoptosis and potent antiproliferative activity against A549 cells. In pharmacokinetic studies, celastrol in the CG-M vector had an extended retention in blood and therefore a potential enhancement of the EPR effect. Collectively, these results suggest that CG-M can be used as stable and controllable released delivery systems of combinational drugs for lung cancer therapy.

## Supplementary Material

IDRD_Ding_et_al_Supplemental_content.docx

## Notes

**Supplementary material available online.**
