# Supplementary material for: Preliminary study on fabrication, characterization and synergistic anti-lung cancer effects of self-assembled micelles of covalently conjugated celastrol–polyethylene glycol–ginsenoside Rh2
Source: Drug Deliv. 2017 May 22;24(1):834–45. doi: 10.1080/10717544.2017.1326540 (PMC8241176; doi:10.1080/10717544.2017.1326540)
Supplement: IDRD_Ding_et_al_Supplemental_content.docx [file IDRD_A_1326540_SM7320.docx]

**Supporting Information**

**Preliminary study on fabrication, characterization, and synergistic anti-lung cancer effects of self-assembled micelles of covalently conjugated celastrol-polyethylene glycol-ginsenoside Rh2**

Peng Li^1,2^, XiaoYue Zhou^1^, Ding Qu^2,3,*^, Mengfei Guo^2,3^, Chenyi Fan^2,3^, Tong Zhou^1^, Yang Ling^1,4,*^

^1^ Department of Oncology, Changzhou Cancer Hospital of Soochow University, Changzhou 213032, P.R. China

^2^ Affiliated Hospital of Integrated Traditional Chinese and Western Medicine, Nanjing University of Chinese Medicine, Nanjing 210028, P.R. China

^3^ Jiangsu Province Academy of Traditional Chinese Medicine, Nanjing, 210028, P.R. China

^4^ Clinical oncology laboratory, Changzhou Cancer Hospital of Soochow University, Changzhou 213032, P.R. China

*Corresponding author. Email: quding1985@hotmail.com (D. Qu); Email: tgpaper@126.com (L. Yang)


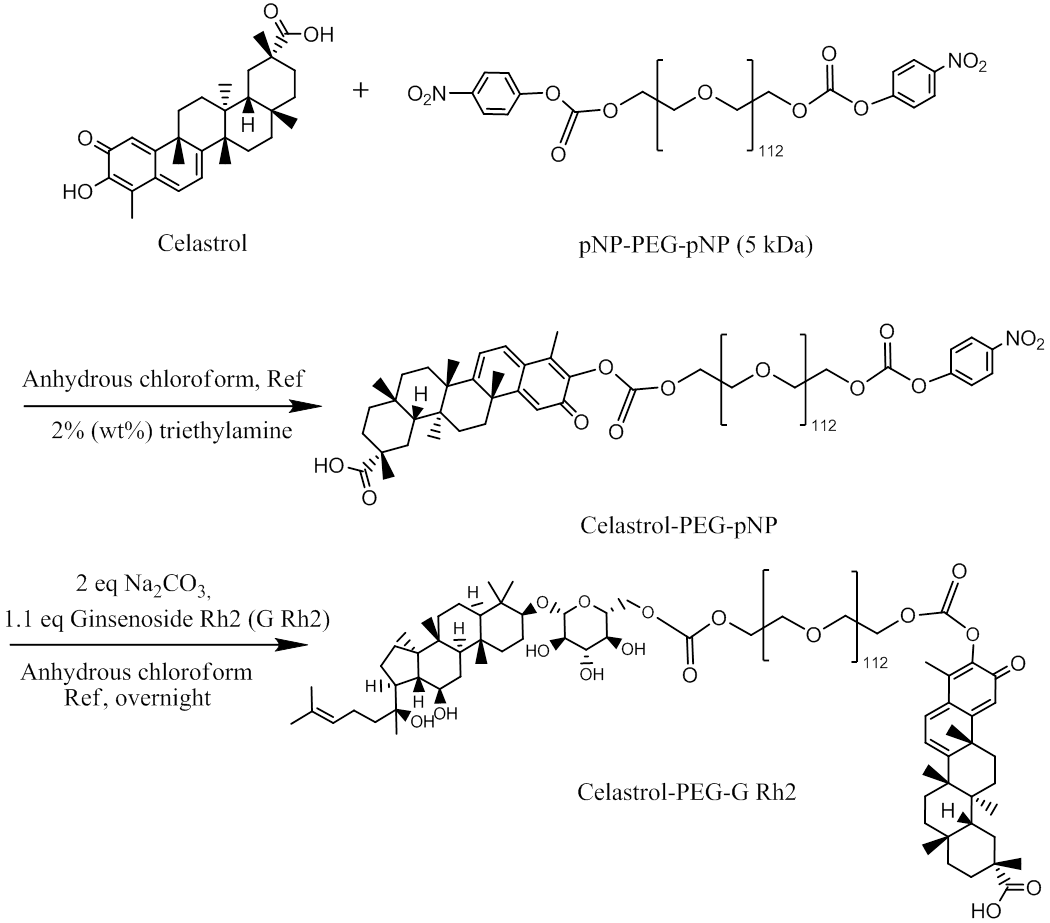


Figure S1. The synthesis of amphipathic polyethylene glycol derivative biterminally modified with celastrol and ginsenoside Rh2 (Celastrol-PEG-G Rh2).


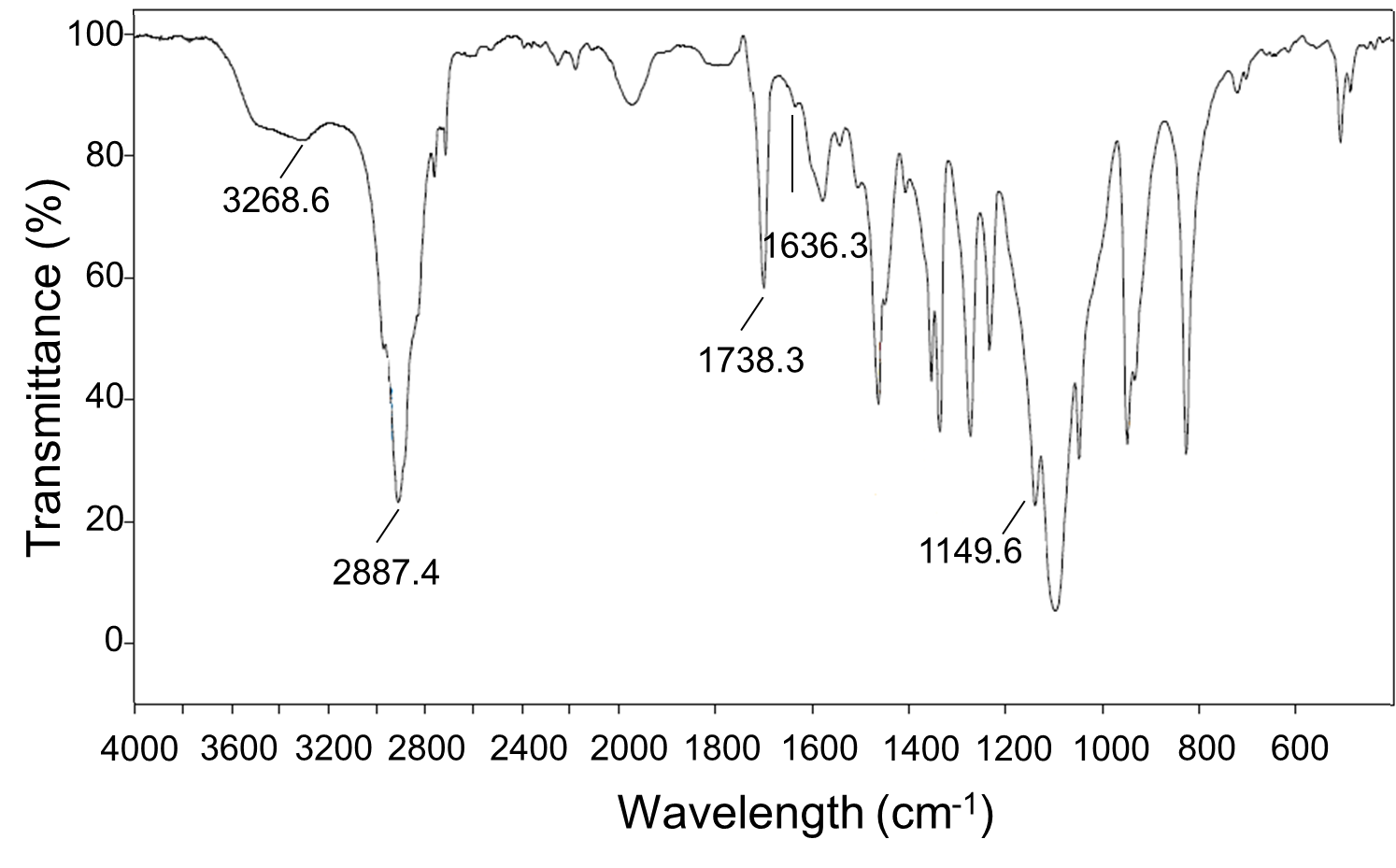


Figure S2. The infrared spectrum of Celastrol-PEG-G Rh2. The signals of several characteristic peaks were labeled.
